# Organic Acid Accumulation and Associated Dynamic Changes in Enzyme Activity and Gene Expression during Fruit Development and Ripening of Common Loquat and Its Interspecific Hybrid

**DOI:** 10.3390/foods12050911

**Published:** 2023-02-21

**Authors:** Honghong Deng, Xuelian Li, Yang Wang, Qiaoli Ma, Yuge Zeng, Yinchun Xiang, Mingmin Chen, Huifen Zhang, Hui Xia, Dong Liang, Xiulan Lv, Jin Wang, Qunxian Deng

**Affiliations:** College of Horticulture, Sichuan Agricultural University, Chengdu 611130, China

**Keywords:** loquat, organic acid, metabolism, gene expression, enzyme activity

## Abstract

Loquats have gained increasing attention from consumers and growers for their essential nutrients and unusual phenology, which could help plug a gap period at market in early spring. Fruit acid is a critical contributor to fruit quality. The dynamic changes in organic acid (OA) during fruit development and ripening of common loquat (Dawuxing, DWX) and its interspecific hybrid (Chunhua, CH) were compared, as well as the corresponding enzyme activity and gene expression. At harvest, titratable acid was significantly lower (*p* ≤ 0.01) in CH (0.11%) than in DWX loquats (0.35%). As the predominant OA compound, malic acid accounted for 77.55% and 48.59% of the total acid of DWX and CH loquats at harvest, followed by succinic acid and tartaric acid, respectively. PEPC and NAD-MDH are key enzymes that participate in malic acid metabolism in loquat. The OA differences in DWX loquat and its interspecific hybrid could be attributed to the coordinated regulation of multiple genes and enzymes associated with OA biosynthesis, degradation, and transport. The data obtained in this work will serve as a fundamental and important basis for future loquat breeding programs and even for improvements in loquat cultural practices.

## 1. Introduction

Common loquat (*Eriobotrya japonica* Lindl.) is an important sub-tropical fruit crop belonging to the tribe *Photinieae*, subfamily Maloideae, and family Rosaceae [1]. Consumers now demand nutritional and health-promoting qualities in fruit and its derivatives. The consumption of loquat fruit has been following an increasing trend, which could be mainly ascribed to its delicious taste and high content of nutritional compounds such as carbohydrates, proteins, vitamins, minerals, carotenoids, phenolics, flavonoids, dietary fibers, phenolic compounds, and organic acids (OAs) [2,3,4]. In addition, its roots, leaves, and flowers have been credited with important medical value due to their anti-inflammatory and antitumor properties. They have long been used to treat inflammation, diabetes, cancer, bacterial infection, aging, pain, and allergy in traditional Chinese medicine [5,6,7,8].

Unlike other temperate fruit crops, common loquat trees bloom in autumn and early winter, and the fruit matures in spring and early summer, when very few fleshy fruits are available at that time in the marketplace [1]. This unusual phenology makes it a popular fruit crop for consumers, and few competitive fruits being in the market makes it a profitable crop for growers. However, common loquat flowers and young fruits are susceptible to low temperature and damage by winter frosts [9,10]. Different from the flowering time of common loquat, bangal loquat (*E. bengalensis* f. Hook.) blooms in spring and is a late-maturing loquat. However, when used as parent in the processing of interspecific hybridization, it shows poor compatibility: the success hybridization rate is less than 10% based on our more than 20 years of loquat-breeding experience.

In our loquat-breeding programs, ‘Chunhua’ (CH), an interspecific hybrid of common loquat (Dawuxing, DWX, *E. japonica* (Thunb.) Lindl.) and bangal loquat (*E. bengalensis* f. Hook.), was recently released [11]. Combining the advantages of parents, CH loquat blooms in spring, thus escaping chilling injuries in winter, and matures almost one month later than common loquat. This late-maturing characteristic of CH is essential to extend the loquat market season when common loquat is no longer in supply. In addition, CH loquat is defined as a low-acid cultivar, while its female parent (DWX) is a moderate-acid cultivar based on more than ten years of observation. DWX is the most popular common loquat cultivar among the commercially available varieties in China. It originated from the seedlings of local loquat and was developed in 1983 by Yongnian Zhou of the Institute of Pomology, Longquanyi District, Chengdu City, Sichuan Province, China.

Sugar, acid, aroma, color, and texture are key components of fruit quality traits determining consumer satisfaction and appreciation. The acidity levels are mostly attributed to the components and contents of OAs [12], particularly the malic and citric acids in fleshy fruits [13]. OAs also contribute to plant growth and development, pH regulation, storage carbon molecule, and stress responses [12,13,14]. The similarity in the genetic origin and difference in fruit acidity between these two important loquat cultivars provide us an excellent opportunity to understand OA metabolism and accumulation in loquat fruits and identify key factors influencing loquat fruit acid.

**Figure 1:**
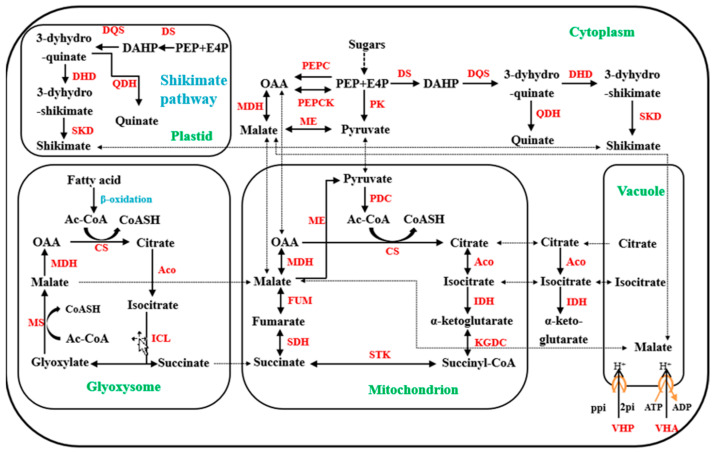
The current known metabolic pathways for organic acids in fleshy fruits. Abbreviations: acetyl coenzyme A, Ac-CoA; aconitase, ACO; coenzyme A, CoSAH; citrate synthase, CS; 3-deoxy-D- arabino-heptulosonate 7-phosphate, DAHP; dehydroquinate dehydratase, DHD; 3-dehydroquinate synthase, DQS; DAHP synthase, DS; erythrose-4-phosphate, E4P; fumarase, FUM; isocitrate lyase, ICL; isocitrate dehydrogenase, IDH; α- ketoglutaric acid dehydrogenase complex, KGDC; malate dehydrogenase, MDH; malic enzyme, ME; malate synthase, MS; oxaloacetic acid, OAA; pyruvate dehydrogenation complex, PDC; phosphoenolpyruvate, PEP; PEP carboxylase, PEPC; PEP carboxykinase, PEPCK; pyruvate kinase, PK; quinate dehydrogenase, QDH; succinate dehydrogenase, SDH; shikimate dehydrogenase, SKD; succinate thiokinase, STK; vacuolar-type H^+^-ATPase, VHA; vacuolar-type H^+^-Ppase, VHP. Dashed arrows indicate organic acid transport.

The current consensus is that the net balance of synthesis, degradation, and transport determines OA accumulation in fruit [13]. Several pathways for malic and citric acid metabolism in fleshy fruits have been characterized [13], as illustrated in Figure 1. Key enzymes involved in malate and citrate biosynthetic pathways are phosphoenolpyruvate carboxylase (PEPC), malate dehydrogenase (MDH), and citrate synthase (CS), while key enzymes involved in OA degradation are phosphoenol carboxykinase (PEPCK), malic enzyme (ME), and aconitase (ACO) [12,14]. Once malic and citric acids have been synthesized in cytosol, their accumulation levels depend largely on the transport from cytosol to vacuole [15]. These complex processes are mediated by numerous vacuolar transporters [13,15], such as tonoplast dicarboxylate transporter (tDT) [16] and channels of aluminum-activated malate transporters (ALMTs) [17]. In addition, the facilitated diffusion of malic and citric acids into vacuole can be caused by tonoplast proton pumps like vacuolar-type H^+^-ATPase (VHA) and vacuolar-type H^+^-PPase (VHP) [18].

A better understanding of the composition, content, and accumulation of OAs is necessary before any genetic improvements or cultural practices can be made. The objectives of this study were to evaluate the composition and content of OAs in key stages of fruit development and ripening of DWX loquat and its interspecific hybrid. The corresponding gene expression and enzyme activity changes concomitant with fruit development and ripening were also explored. The outcomes of this study reveal the physiological processes and molecular mechanisms underlying the OA changes of loquat fruit. The comparisons of the two important cultivars provide an excellent opportunity to identify factors impacting loquat OAs.

## 2. Materials and Methods

### 2.1. Plant Materials and Sampling

This study was conducted using five-year-old (in 2021) bearing loquat trees in fields of the loquat experimental station of College of Horticulture, Sichuan Agricultural University, located at Shimian County, Ya’an City, Sichuan Province, China (29°18’ N, 102°32’ E). The trees were grafted on DWX rootstock with 4 m spacing between rows and 4 m between plants. The row orientation was from north to south. All plants were subjected to identical cultural practices for loquat in this area throughout the experiment, including normal irrigation, fertilization, pest control, and fruit-thinning.

**Figure 2:**
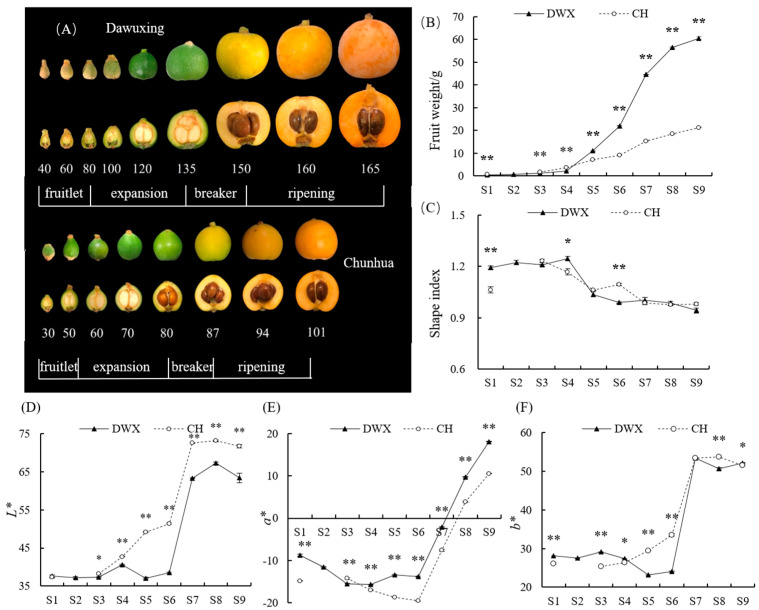
Fruit phenotype (**A**), weight (**B**), shape index (**C**), and color changes (**D**–**F**) during fruit development and ripening of Dawuxing and Chunhua loquats. The x axis in (**A**) presents days after full bloom (DAFB) and the longitudinal section of fruits corresponding to each DAFB are shown in (**A**). The x axes in (**B**–**F**) represent different developmental stages of loquat fruits. The asterisks in the line charts indicate significant differences (*: *p* ≤ 0.05; **: *p* ≤ 0.01).

Four representative stages of loquat fruit development and ripening are the fruitlet (including a lag phase), cell expansion, breaker, and ripening stages according to [19] and our previous observations. CH loquat blooms in spring, so there is no lag phase in young fruit. Specifically, nine sampling points of DWX loquat included 40, 60, 80, 100, 120, 135, 150, 160, and 165 days after full bloom (DAFB), and eight sampling points of CH loquat were 30, 50, 60, 70, 80, 87, 94, and 101 DAFB, as shown in Figure 2A. The time when the whole inflorescence had approximately 75% of the flowers open at full-bloom stage was set as the baseline (0 DAFB). Loquat fruits grow rapidly approximately one and half months before harvest and were thus sampled at a shorter-time interval. Three fruit samples were randomly collected from the upper, middle, and lower canopy of each tree. The fruit samples from five trees (in total fifteen fruits) consisted of one biological replicate. Each stage consisted of three biological replicates.

### 2.2. Chemicals and Reagents

High-performance liquid chromatography (HPLC)-grade regents, including potassium dihydrogen phosphate, phosphoric acid, oxalic acid, tartaric acid, malic acid, α-ketoglutaric acid, citric acid, and succinic acid, were purchased from Sigma-Aldrich (St. Louis, MO, USA). Aqueous solutions were prepared using ultra-purified water (18.2 MΩ. cm) from a Milli-Q gradient water purification system (Millipore Corporation, Bedford, MA, USA).

### 2.3. Fruit Weight, Shape, and Color Measurement

Fruit weight was determined using a 0.01-g sensitive balance and obtained from an average of five fruits of each biological replicate. Fruit length was measured longitudinally from the top to the base of fruit and diameter was determined at the equator of the fruit, cut longitudinally. Dividing the fruit vertical length by its transverse diameter yielded the fruit shape index. A CM-2600d spectrophotometer (Konica Minolta, Tokyo, Japan) was used to determine the fruit color. Five fruits of each biological replicate were randomly selected and transversely sectioned. Three points around the equatorial plane of each fruit were measured. The resulting L*, a*, and b* values indicate lightness, greenness, and yellowness, respectively.

### 2.4. Determination of Titratable Acidity (TA)

Fruit TA was determined by titration to a pH of 8.2 with 0.1 mol L^−1^ NaOH and the results were represented as percentage of malic acid (g of malic acid per 100 g fresh weight (FW)).

### 2.5. Determination of OAs Using HPLC Coupled with Ultraviolet (UV) Detection

Extraction and quantification of OAs were performed using HPLC coupled with UV detection following the previously published protocol by [20] with minor modifications. Briefly, loquat pulp samples were grinded to a fine powder in liquid nitrogen. Then, 0.5 g powder was suspended in 5 mL ultra-purified water, boiled in 80 °C for 1 h, and centrifugated at 8000× *g* for 10 min. The resultant supernatant was filtered through 0.22 μm filter (Millipore Corporation). Finally, 10 μL of sample was injected to an HPLC instrument equipped with an Agilent 1260 Infinity Ⅱ system and a diode array detector (Agilent Technologies Inc., Palo Alto, CA, USA). OA compound separation was achieved using a ZORBOX SB-C18 column (4.6 × 150 mm, 5 μm) (Agilent Technologies Inc.). Elution was carried out with a mobile phase made of 0.04 mol·L^−1^ KH_2_PO_4_-H_3_PO_4_ solution, adjusted to pH 2.3 with H_3_PO_4_. The flow rate was 0.5 mL·min^−1^, column temperature was 35 °C, and UV detection wavelength was 210 nm. OA compounds were identified using authentic standard compounds and calibrated with solution of known concentrations.

### 2.6. Assay of OA Metabolism-Related Enzyme Activity

Crude enzymes were extracted according to the methods described by [21] with some modifications. In brief, the extraction buffer contained 0.2 mol·L^−1^ Tris-HCl (pH8.2), 10 mmol·L^−1^ isoascorbic acid, 5 mmol·L^−1^ MgCl_2_, 2 mmol·L^−1^ ethylenediaminetetraacetic acid (EDTA), 0.1% (*w/v*) bovine serum albumin (BSA), 0.1% (*v/v*) Triton X-100, 10% (*v/v*) glycerol, 0.5 mmol·L^−1^ phenylmethylsulfonyl fluoride (PMSF), and 2% (*w/v*) insoluble polyvinylpolypyrrolidone (PVPP). The reaction mixtures used for each enzyme activity measurement are as follows.

PEPC: 50 mmol·L^−1^ Tris-HCl (pH 8.5), 2 mmol·L^−1^ MgCl_2_, 2 mmol·L^−1^ dithiothreitol (DTT), 0.5 mmol·L^−1^ glutathione (GSH), 0.2 mmol·L^−1^ reduced nicotinamide adenine dinucleotide-disodium salt (NADH-Na_2_), 4 mmol·L^−1^ MnSO_4_, and 2.5 mmol·L^−1^ phosphoenolpyruvate.

Nicotinamide adenine dinucleotide-malate dehydrogenase (NAD-MDH): 50 mmol·L^−1^ Tris-HCl (pH 8.5), 2 mmol·L^−1^ MgCl_2_, 10 mmol·L^−1^ NaHCO_3_, 0.5 mmol·L^−1^ EDTA, 0.5 mmol·L^−1^ GSH, 0.2 mmol·L^−1^ NADH-Na_2_, and 2 mmol·L^−1^ oxaloacetic acid (OAA).

Nicotinamide adenine dinucleotide phosphate-malic enzyme (NADP-ME): 50 mmol·L^−1^ Tris-HCl (pH 7.5), 2 mmol·L^−1^ MnSO_4_, 0.5 mmol·L^−1^ EDTA, 2 mmol·L^−1^ DTT, 0.2 mmol·L^−1^ NADH-Na_2_, and 3 mmol·L^−1^ malic acid.

CS: 50 mmol·L^−1^ Tris-HCl (pH 9.0), 0.05 mmol·L^−1^ 5,5-dithio-bis-(2-nitrobenzoic acid), 0.08 mmol·L^−1^ acetyl coenzyme A, and 2 mmol·L^−1^ OAA.

ACO: 50 mmol·L^−1^ Tris-HCl (pH 7.5), 0.1 mmol·L^−1^ NaCl, 4 mmol·L^−1^ FeSO_4_, 0.2 mmol·L^−1^ NAD, and 20 mmol·L^−1^ citric acid monohydrate.

NAD-isocitrate dehydrogenase (NAD-IDH): 40 mmol·L^−1^ HEPES buffer solution (pH 8.2), 0.5 mmol·L^−1^ EDTA, 4 mmol·L^−1^ MnSO_4_, 0.2 mmol·L^−1^ NAD, and 10 mmol·L^−1^ trisodium hydrogen 3-carboxylato-2,3-dideoxy-1-hydroxypropane-1,2,3-tricarboxylate.

The enzyme activity of CS was measured spectrophotometrically at 412 nm, and the enzyme activities of PEPC, NAD-MDH, NADP-ME, ACO, and NAD-IDH were at 340 nm.

### 2.7. Quantification of Gene Expression Using Quantitative Real-Time Polymerase Chain Reaction (qRT-PCR)

Total RNA from the fruit samples described above was extracted using TRIzol^®^ reagent (Invitrogen, Carlsbad, CA, USA) and treated with TURBO DNA-free^™^ kit (Ambion, Austin, TX, USA) to remove DNA contamination, following the manufacturer’s protocol. Purified RNA was quantified using a Nanodrop 2000 spectrophotometer (Thermo Fisher Scientific, Waltham, MA, USA), and RNA integrity was evaluated using 1% agarose gel electrophoresis. First-strand complementary DNA synthesis was conducted using a reverse transcription kit containing a PrimeScript^TM^ RT reagent kit with gDNA Eraser (Perfect Real Time) (Takara, Dalian, China).

Genes related to OA metabolism were selected according to the loquat whole genome sequence [22]. The internal reference genes for normalization of relative gene expression were obtained from [19]. *PEPC2*, *NAD-MDH*, *NADP-ME2*, *VHA-A*, and *VHP1* were obtained from [23]. *ALTM*s were obtained from [24]. The detailed sequences of primers used are listed in Supplementary Table S1.

qRT-PCR was performed on a CFX96 Touch Real-Time PCR C1000 Thermal Cycler system (Bio-Rad, Hercules, CA, USA) using Brilliant III Ultra-Fast SYBR Green QPCR Master Mix (Agilent Technologies Inc., Santa Clara, CA, USA), following the manufacturer’s recommendations. For each biological replicate, three technical replicates of each PCR were performed.

### 2.8. Statistical Analyses

Means and standard deviation (SD) were calculated using the Microsoft Excel software. Differences within cultivars at each time point were statistically evaluated by one-way analysis of variance (ANOVA) using Tukey’s honest significant difference test with a statistical significance of *p* ≤ 0.05. The association between OA components and other continuous variables, namely, OA metabolism enzyme activities and associated gene expressions, was determined using Person’s correlation coefficient (*p* ≤ 0.05). For qRT-PCR, the 2^−ΔΔCT^ method was used to calculate gene expression levels after normalization to the internal reference gene.

## 3. Results

### 3.1. Comparative Analyses of Fruit Phenotype, Weight, Shape, and Color of Dawuxing Loquat and Its Interspecific Hybrid

The fruit skin color of DWX and CH was changing from green in initial breaker stage gradually to dark orange in appearance (Figure 2A) and as pulp color measurements as the fruit ripened (Figure 2D–F), respectively. The development of DWX and CH fruits encompassed an average period of approximately 165 and 101 days, respectively (Figure 2A). The fruit weight curve of DWX loquat followed a single-sigmoid pattern, with very slow growth in the fruitlet stages (80 DAFB) and a dramatic increase during the expansion stages (80–135 DAFB), followed by a gradual increase afterwards (150–165 DAFB). However, the fruit weight curve of CH loquat exhibited a nonclassical single-sigmoid pattern. At harvest, the fruit weight of DWX loquat (60.54 ± 0.55 g) was significantly higher (*p* ≤ 0.001) than that of CH loquat (21.26 ± 0.08 g) (Figure 2B). Fruit shape index of DWX and CH loquats reached approximately 1.0 after the breaker stage (Figure 2C).

### 3.2. Comparative Analyses of Organic Acid Compositions and Contents of Dawuxing Loquat and Its Interspecific Hybrid

**Figure 3:**
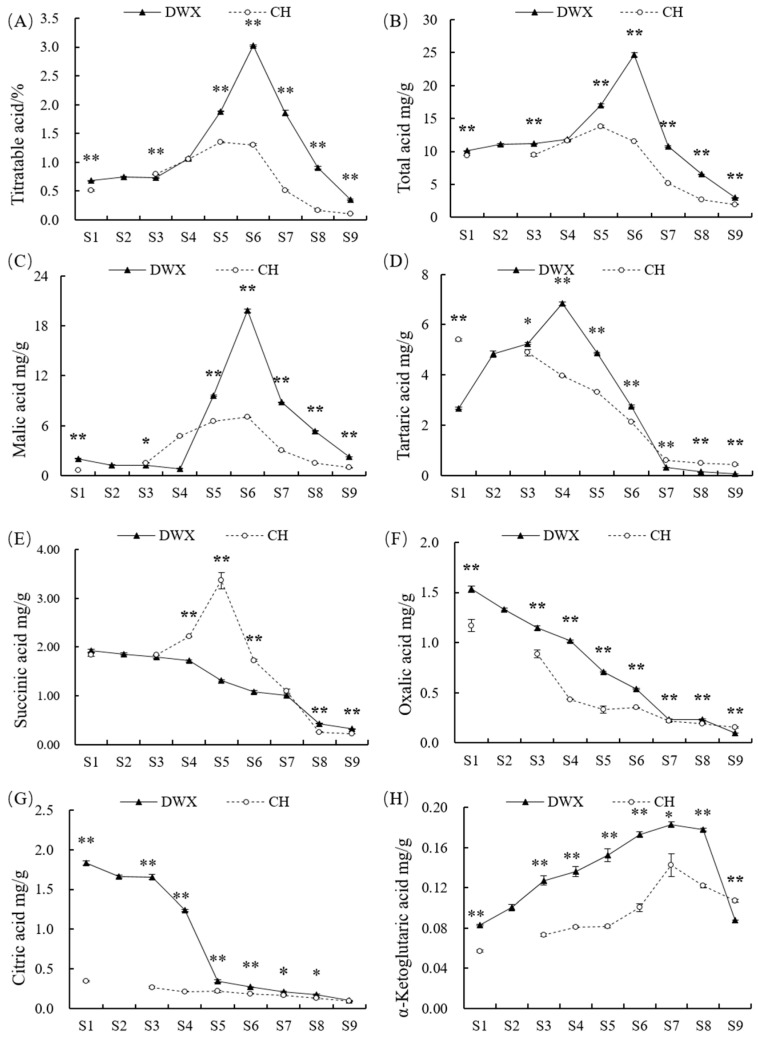
Dynamic changes of titratable acid (**A**), total acid (**B**), malic acid (**C**), tartaric acid (**D**), succinic acid (**E**), oxalic acid (**F**), citric acid (**G**), and α-ketoglutaric acid (**H**) contents at different stages during fruit development and ripening of Dawuxing and Chunhua loquats. The stages on the x-axis corresponded to those presented in Figure 1. The asterisks in the line charts indicate significant differences (*: *p* ≤ 0.05; **: *p* ≤ 0.01).

TA content of DWX loquat remained at a relatively low level at fruitlet stage, then rapidly increased over time, peaking at 135 DAFB (3.02%), and dramatically decreased thereafter. TA content of CH loquat showed a similar trend but peaked at 70 DAFB (1.35%). At harvest, TA was significantly lower (*p* ≤ 0.01) in CH loquat (0.11%) than in DWX loquat (0.35%) (Figure 3A). The dynamics of total acid were similar to that of TA. At harvest, total acid was significantly (*p* ≤ 0.01) lower in CH loquat (1.94 ± 0.03 mg g^−1^ FW) than in DWX loquat (2.94 ± 0.01 mg g^−1^ FW) (Figure 3B).

A total of six OAs, namely, malic acid, tartaric acid, succinic acid, oxalic acid, citric acid, and α-ketoglutaric acid, were detected (Figure 3C–H). Their linear regression equations of standard curves and regression coefficients (R^2^) are listed in Supplementary Table S2. The regression coefficients were generally beyond 0.9995.

The predominant OA compound was malic acid, whose accumulation (Figure 3C) exhibited a similar trend with TA (Figure 3A) and total acid (Figure 3B). The mature CH fruits (0.95 ± 0.02 mg g^−1^ FW) contained significantly lower (*p* ≤ 0.01) levels of malic acid compared to mature DWX fruits (2.28 ± 0.02 mg g^−1^ FW) (Figure 3C).

There was a distinct difference in tartaric acid accumulation between DWX and CH loquats. Tartaric acid content in DWX loquat showed a rapid increase in initial stages, peaking at 100 DAFB, decreased dramatically thereafter until 150 DAFB, and remained at a relatively low level during the subsequent fruit-ripening stages. Differently, it exhibited a continuous decreasing tread in CH loquat until 87 DAFB, followed by a stably low level. At harvest, CH fruits (0.42 ± 0.03 mg g^−1^ FW) contained significantly higher (*p* ≤ 0.01) level of malic acid compared to DWX fruits (0.06 ± 0.00 mg g^−1^ FW) (Figure 3D).

The contents of succinic acid (Figure 3E), oxalic acid (Figure 3F), and citric acid (Figure 3G) exhibited an overall downward trend during fruit development and ripening of the two loquat cultivars, although a unique peak of succinic acid was found at 70 DAFB in CH loquat (3.36 ± 0.16 mg g^−1^ FW). At harvest, CH fruits contained a significantly higher (*p* ≤ 0.01) level of succinic acid (Figure 3E) but a significantly lower (*p* ≤ 0.01) level of oxalic acid (Figure 3F) compared to DWX fruits.

The content of α-ketoglutaric acid increased gradually at fruitlet and expansion stages and reached a maximum value at 160 and 87 DAFB in DWX and CH loquats, respectively, before subsequently declining (Figure 3H). The mature CH fruits contained a significantly higher (*p* ≤ 0.01) level of α-ketoglutaric acid compared to mature DWX fruits.

Tartaric acid was the main OA before expansion stages of these two loquat cultivars, and then malic acid was the predominant OA until ripening stages (Table 1). The largest proportion of tartaric acid in DWX loquat (58.00%) was reported at 100 DAFB, while it plateaued in CH loquat from 30 (57.37%) to 50 (51.58%) DAFB. The proportion of malic acid in DWX loquat reached its maximum value (82.58%) at half-ripe stage (160 DAFB), while that in CH loquat (60.98%) was recorded at breaker stage (80 DAFB) (Table 2). At harvest, tartaric acid (21.79%) and succinic acid (11.22%) had the second largest proportions in CH and DWX loquats, respectively (Table 1).

The correlations between the TA, total acid, and OAs are summarized in Table 2. In this study, we set the threshold for statistically significant correlation between datasets as a *p*-value of less than 0.05 (*p* ≤ 0.05, *) or 0.01 (*p* ≤ 0.01, **). Strongly significantly positive (*p* ≤ 0.01) correlations were found among TA, total acid, and malic acid of the two loquat cultivars. Total acid was significantly positively correlated with tartaric acid in DWX (R^2^ = 0.390 *) and CH (R^2^ = 0.714 **) loquats. α-Ketoglutaric acid had significantly positive correlations with TA (R^2^ = 0.703 **) and total acid (R^2^ = 0.437 *) in DWX loquat, whereas it had significantly negative correlation with total acid (R^2^ = −0.612 **) in CH loquat. Citric acid had a significantly negative correlation with TA (R^2^ = −0.456 *) and a positive correlation with total acid (R^2^ = 0.616 **) in DWX and CH loquat, respectively. Extremely significantly positive correlations were found between succinic acid and TA (R^2^ = 0.866 **) and total acid (R^2^ = 0.950 **) in CH loquat (Table 2).

### 3.3. Comparative Analyses of Enzyme Activity for Organic Acid Metabolism of Dawuxing Loquat and Its Interspecific Hybrid

**Figure 4:**
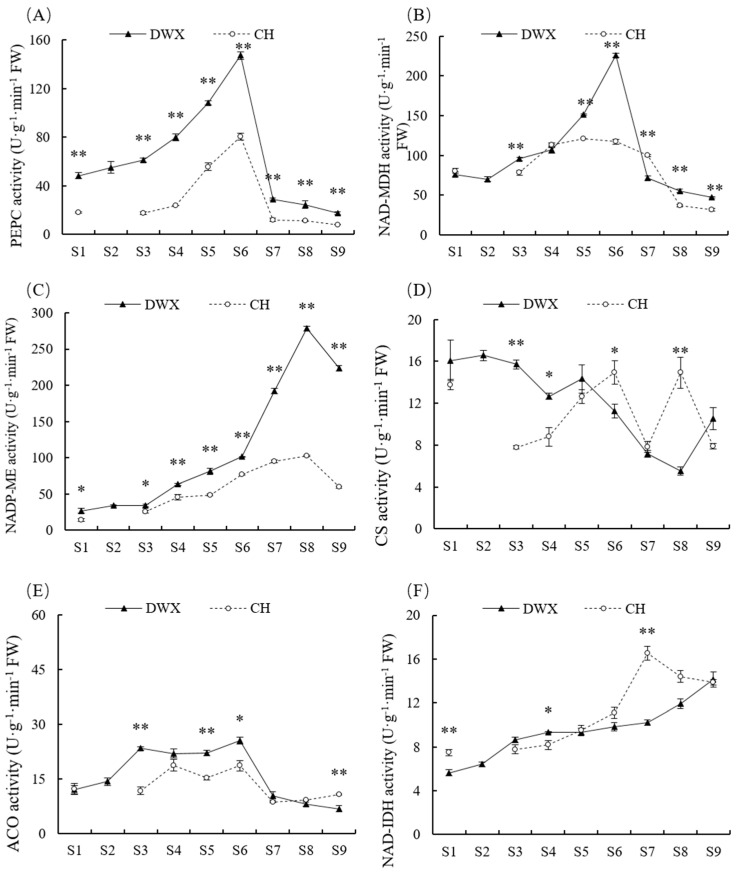
Dynamic changes of (**A**) phosphoenolpyruvate carboxykinase (PEPC), (**B**) NAD-malate dehydrogenase (NAD-MDH), (**C**) NAD-malic enzyme (NAD-ME), (**D**) citrate synthase (CS), (**E**) aconitase (ACO), (**F**) NADP-isocitrate dehydrogenase (NAD-IDH) enzyme activities at different stages during fruit development and ripening of Dawuxing and Chunhua loquats. The stages on the x-axis corresponded to those presented in Figure 1. The enzyme activities were expressed as units of activity per g fresh weight. The asterisks in the line charts indicate significant differences (*: *p* ≤ 0.05; **: *p* ≤ 0.01).

The two loquat cultivars showed a similar dynamic change of PEPC activity during fruit development and ripening, with an initial gradual increase and then a steep drop at breaker stage, followed by a slight decline at the ripening stages (Figure 4A). Although NAD-MDH activity showed a similar trend with PEPC activity, it peaked at 135 DAFB in DWX loquat and plateaued from 60 to 87 DAFB in CH loquat (Figure 4B). NADP-ME activity of DWX and CH loquats increased steadily to a peak at half-ripe stage and then declined at full-ripe stage (Figure 4C). CS activity exhibited an overall downward trend in DWX loquat and fluctuated in CH loquat with two peaks detected at 80 and 94 DAFB (Figure 4D). ACO activity initially increased, then fluctuated in a small range with two peaks detected at the expansion and breaker stages, and gradually stabilized to a low level at ripening stages (Figure 4E). NAD-IDH activity increased continuously until full-ripe stage in DWX loqua, t and it increased continuously to a peak at 87 DAFB in CH loquat (Figure 4F). Significantly higher (*p* ≤ 0.05) enzyme activities of PEPC, NAD-MDH, and NADP-ME were observed across almost all stages in DWX loquat compared to CH loquat (Figure 4).

Malic acid content was strongly positively (*p* ≤ 0.01) correlated with PEPC and NAD-MDH activities in the two loquat cultivars and NAD-IDH activity in CH loquat. α-Ketoglutaric acid content in DWX loquat had significantly positive association with NAD-MDH (R^2^ = 0.405 *) and NADP-ME (R^2^ = 0.423 *) activities and strongly significantly negative association with CS activity (R^2^ = −0.642 **). However, the α-ketoglutaric acid content in CH had a strongly significantly positive association with NADP-ME (R^2^ = 0.908 **) and NAD-IDH (R^2^ = 0.907 **) and had significantly negative association with ACO (R^2^ = −0.420 *). Citric acid had strongly significantly negative (*p* ≤ 0.01) correlations with NADP-ME and NAD-IDH in the two loquat cultivars and significantly positive correlations with CS (R^2^ = 0.751 **) in DWX and NAD-MDH (R^2^ = 0.414 **) in CH. Total acid was extremely significantly positively (*p* ≤ 0.01) correlated with PEPC, NAD-MDH, and ACO activities and positively (*p* ≤ 0.05) correlated with NADP-ME in the two loquat cultivars. An extremely significantly negative (*p* ≤ 0.01) correlation was observed between total acid and NAD-IDH activity (Table 3).

### 3.4. Comparative Analyses of Gene Expression for Organic Acid Metabolism of Dawuxing Loquat and Its Interspecific Hybrid

In total, expressions of forty genes associated with OA metabolism were analyzed using qRT-PCR (Supplementary Figures S1 and S2). The association between OAs and the expressions of related genes are summarized in Table 4.

## 4. Discussion

### 4.1. Characteristics of Fruit Growth and Development in Dawuxing Loquat and Its Interspecific Hybrid

Common loquat trees bloom in the cold autumn and early winter [1]. In total, common fruits take five to six months to mature, depending on the variety and environmental conditions. In this study, there was little increase in mass or volume of young fruit, which is called the lag phase or lag period. After wintering, common loquat fruits expanded rapidly and the fruit growth rate accelerated. Fruit growth tended to be stable after breaker stage (Figure 2B). The fruit development and ripening of DWX loquat (Figure 2B) fitted a classic single sigmoid curve as previously reported in loquats [25].

Bangal loquat was the male parent of CH loquat [11]. Although it can bloom in Sichuan Province, it cannot bear fruit in this area based on our more than ten years of observation. Therefore, the OA dynamic changes of bangal loquat were not investigated in this study. CH loquat blooms in spring, and its fruit growth escaped chilling injury in winter. There was no lag phase between the fruitlet and expansion stages. Therefore, the development and ripening of CH loquat fruit exhibited a nonclassical single-sigmoid pattern (Figure 2B).

### 4.2. Organic Acid Accumulation in Dawuxing Loquat and Its Interspecific Hybrid

Loquat fruits have received constantly increasing attention from the consumers and growers for the nutritional properties and unusual phenology [1]. Fruit acidity is among the most important quality attributes and largely depends on the accumulation of OAs, such as malic, citric, tartaric, and succinic acids [12,13]. The compositions and contents of OAs are important factors directly influencing taste and organoleptic characteristics, namely sourness and flavor [26].

OA composition varies widely in the pulp of different fruit species; however, it is generally accepted that malic and citric acids are the predominant OAs in most ripe fruits [12,13,14]. For example, malic acid is the most predominant OA and contributes significantly to the favorability and palatability of apple [27], peach [28], and loquat [21], while citric acid is the major OA in citrus [29] and passion fruit [30].

In the present study, the malic acid content accounted for 77.55% and 48.59% of the total acid of DWX and CH loquats at harvest, respectively (Figure 3C, Table 1). At harvest, succinic acid (11.22%) and tartaric acid (21.79%) had the second largest proportion in DWX and CH loquats, respectively (Table 1), while the rest, belonging to other OAs, were present in negligible amounts (Figure 3D–G). Similar compositions of malic acid representing 70–80% of the total acid were found in the Jiefangzhong loquat [20]. Variety differences might lead to differences in OA compositions and contents [21]. In this study, all trees were grown under identical natural conditions and received the same horticultural management. Therefore, we demonstrated in our study that the main difference in OA compositions and contents is closely related to the loquat cultivar.

Our results showed that malic acid was not always the predominant OA compound throughout the loquat fruit development and ripening. For example, a high concentration of tartaric acid was observed in the early development stages (Figure 3, Table 2). OA accumulation is accompanied by the synthesis and degradation processes [13]. Because OAs share the same precursor, the phosphoenolpyruvate [12,14], we could hypothesize that phosphoenolpyruvate is preferentially used for other OA synthesis early in loquat fruit development and then for malic acid synthesis later. This reflected the predominance of malic acid as loquat fruits developed and matured. OA accumulated at the early stages of loquat fruit development and decreased during the later stages (Figure 3), which was similar to most studies [21,31,32].

### 4.3. Organic Acid Synthesis, Degradation, and Transport-Related Enzyme Activity and Gene Expression in Dawuxing Loquat and Its Interspecific Hybrid

To elucidate the molecular mechanism underlying the difference of OA metabolism in the two loquat cultivars, correlation analyses between OA accumulation and enzyme activity and relative gene expression were performed and results were divided into positive and negative correlations. OA accumulation in fruits is determined by the balance of biosynthesis, degradation, and vacuolar storage [13,21] and is strongly connected to the activities of multiple metabolic enzymes [12,14].

In the two loquat cultivars, malic acid accumulation was significantly positively correlated with the enzyme activities of PEPC and NAD-MDH (Table 3). Significantly higher (*p* ≤ 0.05) activities of PEPC and NAD-MDH were observed across almost all stages in DWX loquat compared to low activities of them in CH loquat (Figure 4A,B). In addition, malic acid content was positively correlated with the expressions of *PEPC* and *NAD-MDH* in the two cultivars (Table 4). Malic acid is mainly synthesized in the cytosol and is catalyzed by PEPC and NAD-MDH, and cytosolic NADP-ME has been suggested to participate in malic acid degradation [13,21]. In addition, the results agreed that the decreases in these enzyme activities reduced the rate of malic acid synthesis along with fruit maturation [32]. These results here demonstrated that PEPC and NAD-MDH are key enzymes that participate in malic acid metabolism in loquat and the low activities of PEPC and NAD-MDH was one of important reasons for the low malic acid content in CH loquat.

In the first step of the tricarboxylic acid (TCA) cycle, citric acid is synthesized through the condensation of acetyl-CoA with oxaloacetate, which is catalyzed by CS [29,33]. Overall, citric acid of the two cultivars accumulated in young loquat fruits and decreased along with fruit ripening (Figure 3G). The changes of citric acid content in CH loquat were partly consistent with the activity of CS, because two unique peaks at 80 and 94 DAFB were observed (Figure 4D). Citric acid dynamics in DWX loquat were significantly positively correlated with CS activity (Table 3), demonstrating the contribution of CS enzyme in citric acid biosynthesis in DWX loquat.

These differences in the OA content between the two cultivars may be attributable to the difference in activities of multiple enzymes (Table 3). Citric acid degradation is first catalyzed by ACO into isocitrate via the intermediate product cis-aconitate and then metabolized into 2-oxoglutarate by NADP-IDH [29]. NADP-ME has been suggested to be involved OA degradation during fruit ripening of several species [13,21]. A few studies reported the promoting role of NAD-IDH in citric acid decomposition [34]. Citric acid changes during fruit development, and ripening of *Actinidia eriantha* had no contrary trends and correlations with the NAD-IDH [35]. A positive correlation occurred between ME activity and citric acid content in melon fruit development and ripening and ME activity increased over time [36]. In this study, a significantly negative correlation between citric acid and NAD-IDH and NADP-ME (Table 3) supported that they are key enzymes for citric acid decomposition in loquat. Additionally, NAD-IDH and NADP-ME activities were significantly positively correlated with α-ketoglutaric acid accumulation (Table 4).

Total acid content was significantly highly positively (*p* ≤ 0.01) correlated with the expressions of *PEPC*, *PEPC*2, *NAD-MDH*, *ATP-CS*, *ATP-CS α1*, *VHA-C*, ALMT1, *ALMT12*, and *ALMT14* and negatively (*p* ≤ 0.01) correlated with the expressions of *NADP-ME2*, *ALMT3*, and *ALMT 11* in the two cultivars (Table 4). *VHA-C*, and *VHA-F* belonged to the vacuolar H^+^-ATPase family and had the capability of maintaining the acid pH by a proton pump and participating in transportation across the tonoplast [37]. *ALMTs* are aluminum-activated malate transporters and involved in diverse physiological processes [38]. In summary, the difference in DWX loquat and its interspecific hybrid could be attributed to the coordinated regulation of multiple genes and enzymes associated with distinct stages of OA metabolism.

## 5. Conclusions

Fruit acid is a critical contributor to fruit quality. In this study, the dynamic changes in fruit OA content during the fruit development and ripening stages of DWX loquat and its interspecific hybrid were compared, as well as the corresponding enzyme activity and gene expression. OA accumulated at the early stages of loquat fruit development and decreased during the later ripening stages. TA was significantly lower (*p* ≤ 0.01) in CH loquat than in DWX loquat. Malic acid was the predominant OA compound at fruit ripening stages, followed by succinic acid and tartaric acid in DWX and CH loquats, respectively. PEPC and NAD-MDH are key enzymes that participate in malic acid metabolism in loquat, and the low activities of PEPC and NAD-MDH was one of the important reasons for the low malic acid content in CH loquat. The difference in DWX and its interspecific hybrid could be attributed to the coordinated regulation of multiple genes and enzymes associated with distinct stages of OA metabolism. The data obtained here serves as an important foundation for future genetic improvements or cultural practices.

## Figures and Tables

**Table 1:** The proportions of organic acids at different stages of fruit development and ripening of Dawuxing and Chunhua loquats.

Stage ^a^	Oxalic Acid		Tartaric Acid		Malic Acid		α-Ketoglutaric Acid		Citric Acid		Succinic Acid	
	DWX	CH	DWX	CH	DWX	CH	DWX	CH	DWX	CH	DWX	CH
S1	15.21	12.43	26.52	57.37	20.23	6.41	0.82	0.61	18.17	3.64	19.05	19.55
S2	12.06		43.64		11.52		0.91		15.06		16.81	
S3	10.28	9.37	46.91	51.58	10.83	16.08	1.14	0.77	14.83	2.80	16.01	19.41
S4	8.61	3.75	58.00	34.06	7.17	40.60	1.15	0.70	10.50	1.81	14.57	19.07
S5	4.16	2.40	28.64	24.01	56.57	47.16	0.89	0.59	2.02	1.55	7.70	24.28
S6	2.19	3.05	11.17	18.54	80.48	60.98	0.70	0.90	1.09	1.60	4.37	14.93
S7	2.20	4.16	2.93	11.36	81.92	57.64	1.70	2.58	1.96	3.16	9.29	21.09
S8	3.57	7.17	2.02	17.97	82.48	56.03	2.73	4.55	2.61	4.76	6.59	9.52
S9	3.33	8.13	1.99	21.79	77.51	48.59	2.99	5.75	3.42	4.52	10.76	11.22

Note: ^a^ The stage numbers in this column corresponded to those presented in Figure 1A. Abbreviations: Dawuxing, DWX; Chunhua, CH.

**Table 2:** Correlations between the titratable acid, total acid, and organic acids.

Index	Titratable Acid		Total Acid	
	Dawuxing	Chunhua	Dawuxing	Chunhua
titratable acid	1	1	0.883 **	0.940 **
oxalic acid	−0.269	0.093	0.160	0.383
tartaric acid	0.005	0.457 *	0.390 *	0.714 **
malic acid	0.948 **	0.886 **	0.789 **	0.704 **
α-ketoglutaric acid	0.703 **	−0.366	0.437 *	−0.612 **
critic acid	−0.456 *	0.348	−0.054	0.616 **
succinic acid	−0.087	0.866 **	0.301	0.950 **

Note: * and ** indicate that the correlation coefficients are significant at 0.05 and 0.01 levels, respectively. The values without asterisks denote *p* > 0.05.

**Table 3:** Correlation analysis of organic acids with related enzyme activities.

Index		PEPC	NAD-MDH	NADP-ME	CS	ACO	NAD-IDH
malic acid	DWX	0.676 **	0.808 **	0.167	−0.308	0.369	0.169
	CH	0.570 **	0.756 **	0.251	−0.247	0.224	0.793 **
α-ketoglutaric acid	DWX	0.298	0.405 *	0.423 *	−0.642 **	0.228	0.287
	CH	−0.177	−0.225	0.908 **	−0.105	−0.420 *	0.907 **
citric acid	DWX	−0.020	−0.176	−0.817 **	0.751 **	0.234	−0.805 **
	CH	0.098	0.414 *	−0.770 **	0.159	0.200	−0.766 **
total acid	DWX	0.947 **	0.960 **	−0.435 *	0.200	0.794 **	−0.314
	CH	0.693 **	0.872 **	−0.505 **	0.202	0.730 **	−0.731 **

Note: * and ** indicate that the correlation coefficients are significant at 0.05 and 0.01 levels, respectively. The values without asterisks denote *p* > 0.05.

**Table 4:** Correlation analysis of organic acids with related genes expression.

Genes	Malic Acid		α-Ketoglutaric Acid		Citric Acid		Total Acid	
	DWX	CH	DWX	CH	DWX	CH	DWX	CH
*PEPC*	0.865 **	0.753 **	0.532 **	−0.019	−0.567 **	0.178	0.647 **	0.657 **
*PEPC2*	0.275	0.198	−0.189	−0.685 **	0.478 *	0.624 **	0.726 **	0.692 **
*NAD-MDH*	0.792 **	0.578 **	0.545 **	−0.453 *	−0.262	0.499 *	0.707 **	0.820 **
*NADP-ME*	0.235	−0.215	0.568 **	−0.536 **	−0.540 **	0.458 *	−0.002	0.283
*NADP-ME2*	−0.186	−0.495 *	−0.303	0.664 **	−0.486 *	−0.742 **	−0.623 **	−0.925 **
*NADP-ME4*	0.376	−0.176	0.765 **	−0.205	−0.637 **	−0.046	0.109	−0.051
*CS*	−0.271	0.769 **	−0.126	−0.315	0.675 **	0.199	0.152	0.801 **
*ATP-CS α1*	0.711 **	0.557 **	0.355	−0.194	−0.229	0.160	0.873 **	0.497 *
*ATP-CS α2-1*	0.477 *	−0.030	0.825 **	0.321	−0.468 *	−0.189	0.187	−0.219
*ATP-CS β2*	0.388 *	0.442 *	0.322	−0.208	−0.055	0.199	0.591 **	0.402
*ACO*	0.300	0.457 *	0.675 **	−0.767 **	−0.596 **	0.616 **	−0.097	0.858 **
*NAD-IDH1*	0.707 **	−0.053	0.874 **	−0.380	−0.547 **	0.421 *	0.490 **	0.299
*NAD-IDH5*	0.436 *	0.228	0.793 **	0.360	−0.454 *	−0.027	0.124	0.070
*tDT2*	0.014	−0.383	−0.259	0.616 **	0.394 *	−0.250	0.108	−0.601 **
*VHA-A*	0.188	0.094	0.520 **	0.021	0.019	0.201	0.371	0.186
*VHA-A3*	0.823 **	−0.556 **	0.624 **	−0.515 *	−0.380	0.628 **	0.681 **	−0.012
*VHA-B2-1*	0.501 **	0.226	0.843 **	−0.080	−0.510 **	0.171	0.240	0.338
*VHA-B2-2*	−0.420 *	0.119	−0.480 *	0.301	−0.035	−0.224	−0.556 **	−0.119
*VHA-C*	−0.073	−0.104	−0.138	−0.630 **	0.633 **	0.766 **	0.456 *	0.462 *
*VHA-D2*	0.491 **	−0.242	0.766 **	−0.364	−0.435 *	−0.043	0.194	−0.061
*VHA-E1*	−0.288	−0.275	0.323	−0.171	0.163	0.369	−0.334	0.108
*VHA-F*	−0.306	−0.451 *	−0.103	−0.498 *	0.618 **	0.569 **	0.174	0.104
*VHP1*	0.259	−0.584 **	0.315	0.254	−0.815 **	−0.181	−0.285	−0.633 **
*ALMT1*	0.159	0.027	0.175	−0.824 **	0.148	0.778 **	0.571 **	0.673 **
*ALMT2*	−0.483 *	−0.347	−0.097	−0.286	0.574 **	0.123	−0.137	−0.051
*ALMT3*	−0.150	−0.473 *	0.295	0.472 *	−0.375	−0.486 *	−0.567 **	−0.735 **
*ALMT4*	−0.588 **	−0.251	−0.477 *	−0.348	0.748 **	0.367	−0.140	0.165
*ALMT5*	0.056	−0.242	−0.374	−0.366	0.509 **	0.383	0.298	0.167
*ALMT6*	0.183	−0.800 **	0.081	−0.127	0.011	0.261	0.019	−0.460 *
*ALMT7*	0.444 *	0.352	0.572 **	−0.216	−0.715 **	0.120	0.096	0.473 *
*ALMT8*	0.700 **	−0.405 *	0.855 **	−0.001	−0.652 **	−0.466 *	0.338	−0.513 *
*ALMT9*	−0.078	−0.489 *	0.283	−0.683 **	0.233	0.818 **	0.121	0.208
*ALMT10*	0.580 **	−0.345	0.089	−0.736 **	0.233	0.896 **	0.771 **	0.335
*ALMT11*	0.003	−0.469 *	0.471 *	0.745 **	−0.533 **	−0.775 **	−0.474 *	−0.940 **
*ALMT12*	−0.070	0.072	0.117	−0.830 **	0.605 **	0.786 **	0.416 *	0.684 **
*ALMT13*	−0.193	−0.220	−0.032	−0.805 **	0.573 **	0.827 **	0.223	0.481 *
*ALMT14*	0.405 *	−0.088	0.068	−0.857 **	0.331	0.873 **	0.619 **	0.631 **
*ALMT15*	0.401 *	−0.243	0.806 **	−0.332	−0.398 *	0.322	0.138	0.130
*ALMT16*	0.338	−0.398	−0.131	−0.686 **	0.267	0.847 **	0.623 **	0.245
*ALMT17*	−0.351	−0.579 **	−0.654 **	0.429 *	0.493 **	−0.423 *	−0.233	−0.775 **

Note: * and ** indicate that the correlation coefficients are significant at 0.05 and 0.01 levels, respectively. The values without asterisks denote *p* > 0.05.

## Data Availability

The data and materials supporting the conclusions of this study are included within the article.

## Supplementary Materials

The following supporting information can be downloaded at https://www.mdpi.com/article/10.3390/foods12050911/s1: Figure S1: Dynamic changes in expressions of forty genes related to organic acid metabolism during different stages of fruit development and ripening in Dawuxing and its interspecific hybrid. The stages on the x-axis correspond to those presented in Figure 1. Values are means ± SD of three biological replicates. Figure S2: Figure S1 continued. Table S1. Primers used for quantitative real-time polymerase chain reaction in this study. Table S2. Authentic standard organic acid compounds used in HPLC analysis and their equations of standard curves.
