# Supplementary material for: Organic Acid Accumulation and Associated Dynamic Changes in Enzyme Activity and Gene Expression during Fruit Development and Ripening of Common Loquat and Its Interspecific Hybrid
Source: Foods. 2023 Feb 21;12(5):911. doi: 10.3390/foods12050911 (PMC10000456; doi:10.3390/foods12050911)
Supplement: Supplementary file 1 [file foods-12-00911-s001.zip › foods-2115389-supplementary.pdf]

Table S1. Primers used for quantitative real-time polymerase chain reaction in this study

Gene	Primer F	Primer R	Gene ID in loquat genome or reference
Actin	AATGGAACTGGAATGGTCAAGGC	TGCCAGATCTTCTCCATGTCATCCCA	
PEPC2	GTGGTTCTTGGTAATGTAA	GAAGTGTCATAGCAATCC	
NAD-MDH	CTGGTGTCTTCTATGATA	TCTCTTCTGGATAAGTTC	
NADP-ME2	GAATACCATCCGACAATA	ACATTATCAACGAGAAGT	-
VHA-A	TGAAGGAGGTGATAGACA	ACTGCGGACATTATTATACT	
VHP1	CATCTTCGTTGGTGTATT	TTGCCTGTGTTATATGTG	
PEPC	GTGATGTTCCCAAGACTGAA	TCTCAAACAATGGAACAACC	*Ej00033952*
NADP-ME	GAGTGTCTCGGTTGCTAGTG	ACTTCTGCAGTGGAACTTGA	*Ej00091189*
NADP-ME4	ACACCACAACAAAGGTCTTG	ACTTCTGCAGTGGAACTTGA	*Ej00076553*
CS	TTCCAGAATGCCAGAAAGTA	ACTACAGCACGAGTCCTCAA	*Ej00032441*
ATP-CS α1	GTGAAGCCTGACATGTTGTT	AACTCTTCATTGTGGGGAAT	*Ej00011370*
ATP-CS α2-1	GTGAGCTTTTCAGAGTGTGG	CTGGAATACAGCAAATACGC	*Ej00094058*
ATP-CS β2	TGGAAACTGCTATCAAGGAA	GTTCCTCACCTCTGTCATCA	*Ej00074816*
ACO	ATATCTTGGACGGGTTGTTT	TTCAGCTTCAATACCTCCAA	*Ej00088565*
NAD-IDH1	AACATTATGAAGCTGGCTGA	AACGAGTTGCATACAGCAAT	*Ej00095950*
NAD-IDH5	AGTGGTGAGAGGTGTTGTTG	GCTTTGTGAATTGCAGAGAC	*Ej00051965*
tDT2	GAAGTTGATGGACTGGAACA	GGGCTTTCGATAAAATGTCT	*Ej00066741*
VHA-A3	TGTTTGTTACTTGCGACCTT	AACGGGACAGAGAAGAACTC	*Ej00009754*
VHA-B2-1	GACAACTTTGCCATTGTGTT	TCAATTGTAGGGTCATTTGC	*Ej00095876*
VHA-B2-2	AAAATCCCACTTTTCTCTGC	AATCCCGTTTAAAGAACTGC	*Ej00010503*
VHA-C	AGAACAAGTGCTCGTGAAAA	AAACCTCCCCATAACTGGTA	*Ej00016657*
VHA-D2	CGACAAGGCATTTTATGAAG	AGATTCCTGATCTCCTGCTC	*Ej00017543*
VHA-E1	TCTGCTGAGGAAGAATTCAA	TTCATGGAGTTAACCACGTC	*Ej00045917*
VHA-F	TTTACAGCAAAGGACGACAT	GATGCCACAGATTCAGTGTT	*Ej00074402*
ALMT1	TGATGCAGTCGATCGAAGAG	TGGTCCAAACTTGGAAGGAG	*EVM0037970.1*
ALMT2	AAAGGGTAGGATGCGAAGGT	AATCTCCCAGCTTTCCGAAT	*EVM0000569.1*
ALMT3	GCGGTGATAACCGTGGTAGT	AAAGATCCCAAGCACAATGG	*EVM0022757.1*
ALMT4	GTGGGAGCCTAGACATGGAA	TTTGTTGCTTTGGATGGTCA	*EVM0025487.1*

ALMT5	CTAGCATCCCTGGCACATTT	CGAATTCTCACTTCCGGGTA	*EVM0013481.1*
ALMT6	GCCGCAGAACTGGTAAGAAG	GGTGACATCGGAGAAGGTGT	*EVM0010307.1*
ALMT7	GACTTGGGCTTCAACAGCTC	TTTTCGAGGATCCGAATGAC	*EVM0008191.1*
ALMT8	GGAGCTCCAGAGAGTTGGTG	TTCCCTGGGACGTACTTCAG	*EVM0037785.1*
ALMT9	AGTACGGCTTTCGGGTTTTT	CAGATCCTCTCCCGACCATA	*EVM0008737.1*
ALMT10	TGGGAAAGCATTGAAGGAAC	GTGTGCCAGGGATGCTAGTT	*EVM0028408.1*
ALMT11	GGAAGGTTTTGGGGATGAAT	GGTGAGGTTTCCGATCTTGA	*EVM0021601.1*
ALMT12	TGTGATAGTGCCCGAATTGA	CCAGCAAGCTTTCCAAGTTC	*EVM0017728.1*
ALMT13	AACTATTCCGGCAGATGTGG	ACTAGGGCAACTCCCACCTT	*EVM0022795.1*
ALMT14	GAAGTTCTTGAGGCCACAGC	CAATCCTCCCCAACTCTTCA	*EVM0016148.1*
ALMT15	GGTGCCATGATCTTCATCCT	TGAAATAATCCGCCACACAA	*EVM0012726.1*
ALMT16	TATTGAACGCGGATGATGAA	AAACACCTGTGGGCAAGTTC	*EVM0012851.1*
ALMT17	CGATGGTATTGGTGTTGCAG	AAAGATCCCAAGCACAATGG	*EVM0040195.1*

Table S2. Authentic standard organic acid compounds used in the HPLC analysis and equations of standard curves.

Compound name	Elute time (min)	Linear equation	Correlation coefficient (R^2)
oxalic acid	2.84	y=14.191x-24.94	0.9995
tartaric acid	3.34	y=2.1536x+10.266	0.9999
malic acid	4.14	y=1.7876x+0.1158	1.0000
α-ketoglutaric acid	5.39	y=8.5712x-27.732	1.0000
citric acid	8.25	y=1.0832x-2.0558	1.0000
succinic acid	9.30	y=0.6751x-13.784	0.9996

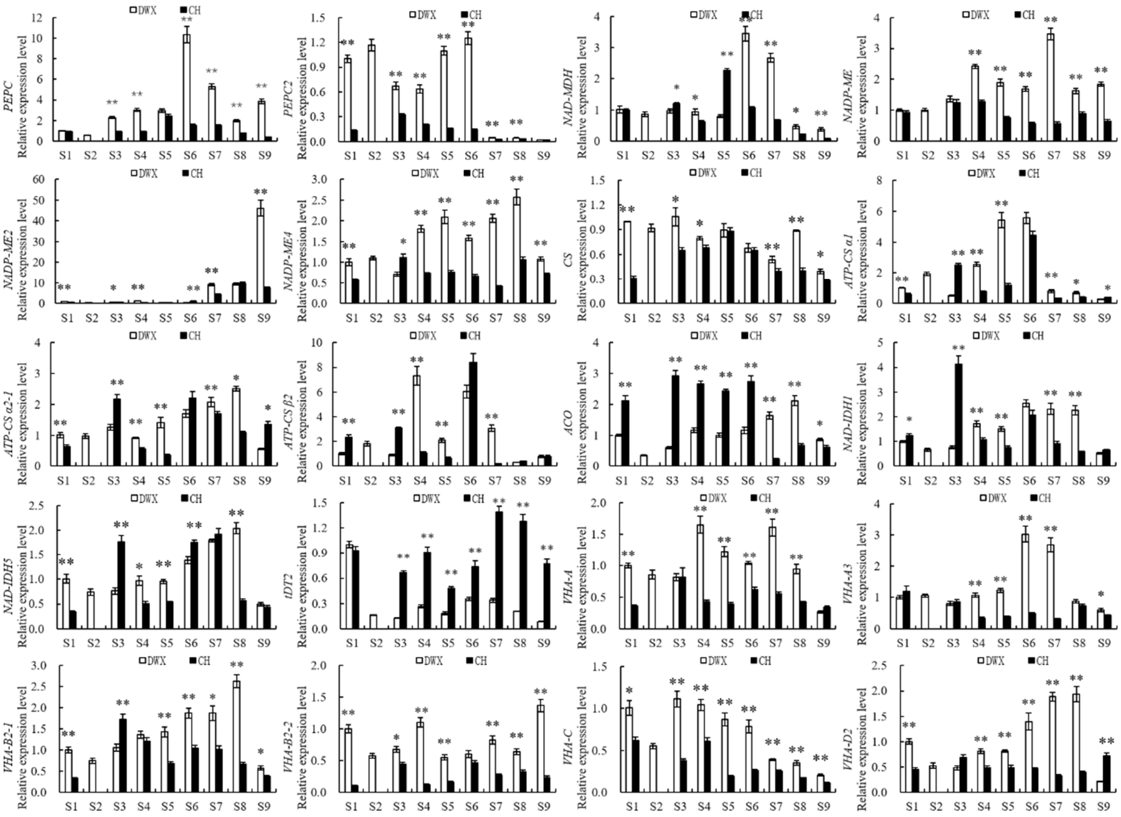

Figure S1. Dynamic changes in expressions of forty genes related to organic acid metabolism during different stages of fruit development and ripening in Dawuxing and its interspecific hybrid. The stages numbered on the x-axis correspond to those presented in Figure 1. Values are means ± SD of three biological replicates. The asterisks indicate significant differences (*: $p \leq 0.05$; **: $p \leq 0.01$).

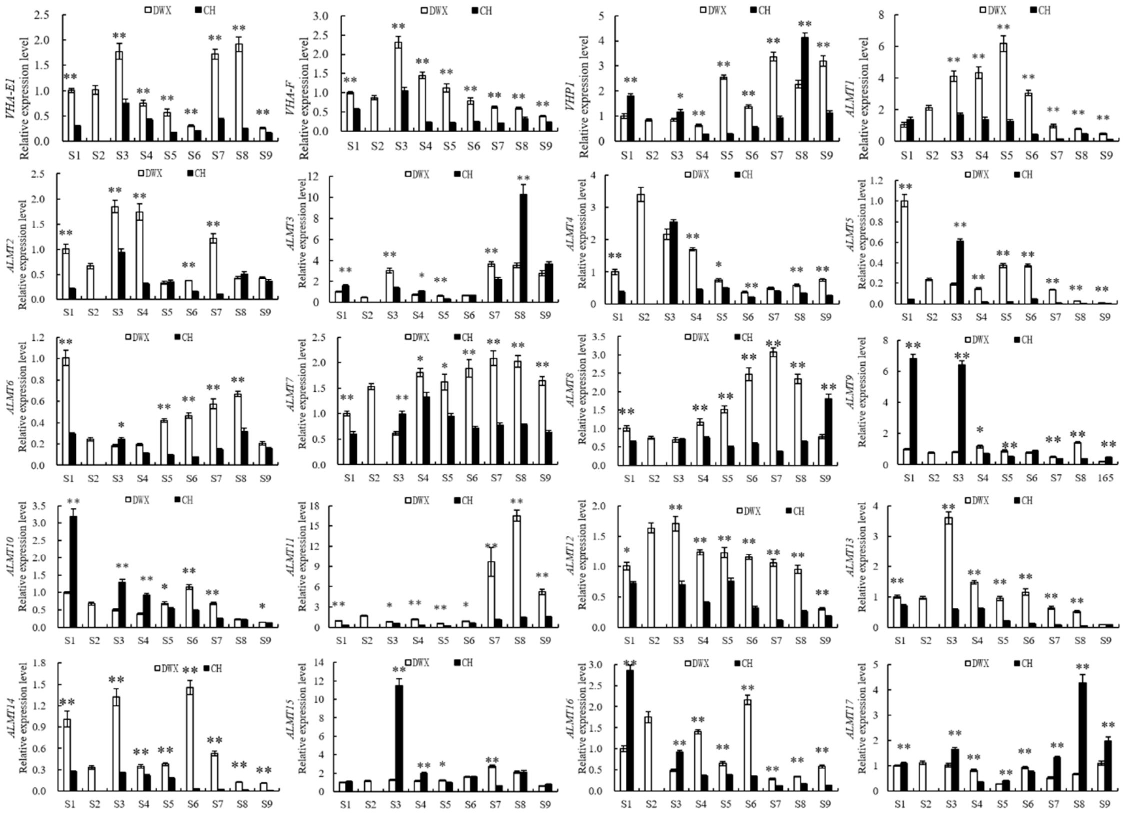

Figure S2: Figure S1 continued.
